# Tibial Tubercle Avulsion Fracture in Young Athletes Surgically Treated: Mid-Long Term Result and Comparison

**DOI:** 10.3390/children12050533

**Published:** 2025-04-22

**Authors:** Lorenzo Moretti, Carlo Amati, Alessandro Geronimo, Andrea Michele Abbaticchio, Maria Paola Miolla, Daniela Dibello, Giuseppe Solarino

**Affiliations:** 1 Orthopaedic & Trauma Unit, Department of Basic Medical Sciences, Neuroscience and Sense Organs, School of Medicine, University of Bari Aldo Moro, AOU Consorziale Policlinico, 70124 Bari, Italy; 2 Orthopaedic & Trauma Unit, Giovanni Paolo XXIII Pediatric Hospital, University of Bari “Aldo Moro” Via Giovanni Amendola, 207, 70126 Bari, Italy

**Keywords:** tibial tubercle avulsion, young athletes, surgery treatment

## Abstract

**Objectives**: Tibial apophysis avulsion fracture is an unusual injury in young pediatric athletes. The mechanism of injury is often related to sports (i.e football and basketball). Fifteen patients who had this kind of fracture underwent surgical Open Reduction and Internal Fixation (ORIF) with two or three cannulated screws and tendon stripping. In this kind of injury and treatment, one of the most important concerns is the recovery of the extensor apparatus strength of the lower limb. **Materials and Methods**: We followed up the patients for 12 months, performing biomechanical tests and a kinetic analysis to evaluate the activation of the leg muscles such as rectus femoris, vastus medialis, vastus lateralis, and semitendinosus muscle fibers and compared them with the contralateral healthy limb. **Results**: The results showed that there was an almost complete recovery of muscle strength activation without any statistically significant differences compared to the healthy limb. **Conclusions**: This surgical treatment appears to be safe and effective in the treatment of tibial apophysis fractures in young athletes, since this type of surgical treatment does not compromise the recovery of the extensor apparatus strength and/or return to sport of the lower limb in case of tibial apophysis fractures in young athletes.

## 1. Introduction

Avulsion fracture of the tibial apophisis is an unusual injury in young patients [1]. The incidence of all growth plate lesions is less than 1%, with 0.25 to 2.7 cases per year [1,2]. Avulsion fractures of the tibial tuberosity affect adolescents, predominantly males, when the proximal tibial physis is about to close and patients usually have strong thigh muscles [1].

The mechanism of injury is often related to sports such as basketball and football, where kids are requested to jump and kick. An increased incidence has been observed in recent years, related to the greater involvement of children in sports. Two main mechanisms of lesion are described in literature: in a movement like jumping, there is first the powerful tension of the extensor muscles with the foot fixed on the floor, and second the knee flexion with the quadriceps contraction when landing on the floor [1,3,4].

During the growth period, epiphysis is 2 to 5 times weaker than the surrounding connective tissues that make up tendons and ligaments, and is therefore more prone to injury [2,5].

Tibial tubercle avulsion injuries in an adolescent whose epiphyseal plates have not yet closed should absolutely not be underestimated, especially with regard to long-term complications such as potential leg length discrepancy or axial deviations, which must be absolutely avoided [6,7].

In this study we collected 15 athlete patients who were surgically treated for an acute avulsion fracture of the tibial tubercle and patellar tendon tear occurred during sport activity. All patients were treated with the same surgical technique and by the same surgeon in the period from January 2020 to July 2023 with a follow up of one year. All the patients followed the same rehabilitation protocol during the first 12 weeks. After that period, patients returned to their normal sports activities.

The purpose was to evaluate the surgical treatment in terms of muscle symmetries and strength by comparing the involved knee to the healthy one in young adolescents athletes with a biomechanical analysis.

## 2. Materials and Methods

### 2.1. Objective

The aim was to compare strength, pain, and function of the knee involved in surgery after the avulsion fracture of tibial apophysis to the contralateral in active and sporty patients performing a biomechanical kinetic analysis. We also evaluated the satisfaction of the patients and the time to return to playing sport and normal activities. This is a retrospective cross-sectional observational study.

### 2.2. Study Population

In this study, 15 competitive young athlete patients were recruited after undergoing surgical treatment for the fracture of the tibial apophysis with patellar tendon rupture due to a trauma during a sport activity.

In total, 10 patients were football players, 3 were basketball players, 1 was a volleyball player, and 1 was a professional dancer (Table 1).

We defined the fractures using the Odgen Classification system (8 type 3, 4 type 2, 2 type 1b, and 1 type 5) [3].

The return to sport was evaluated by TEGNER Activity Scale [8].

*Lysholm knee score* was calculated in all patients after surgery in order to evaluate function and activity in term of stability [9]. All patients have been also tested with the *Physical Component Summary* (PCS) and Mental Component Summary (MCS) to evaluate physical and mental state, as shown in Table 1.

The inclusion criteria were: (1) age between 13 and 17 years old; (2) practice of competitive sporting activity.

Exclusion criteria were the presence of associated or previous articular lesions.

Each participant provided written informed permission.

### 2.3. Surgical Treatment and Complications

**Figure 1:**
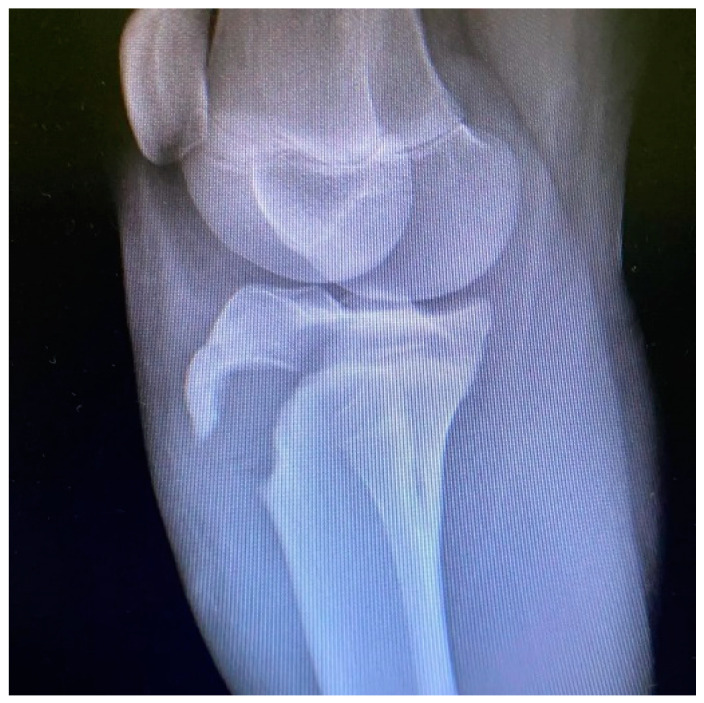
Pre-operative X rays (lateral view).

**Figure 2:**
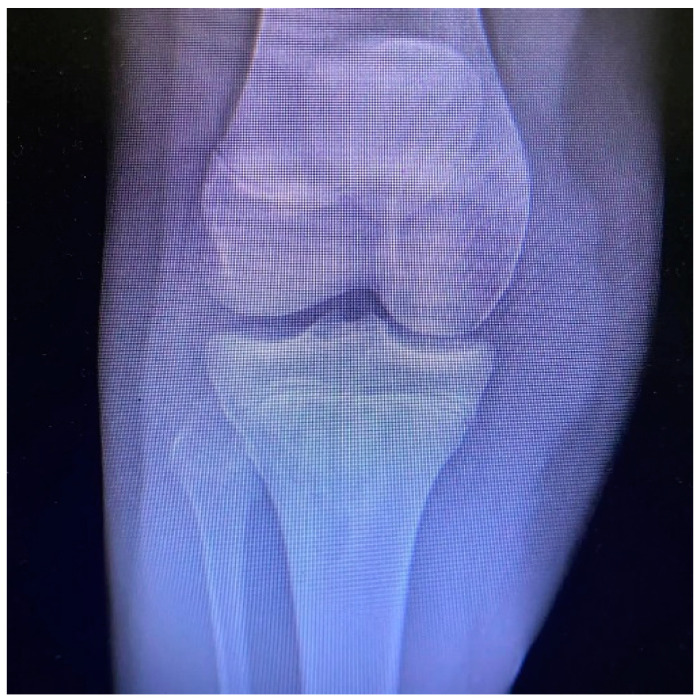
Pre-operative X rays (antero-posterior view).

**Figure 3:**
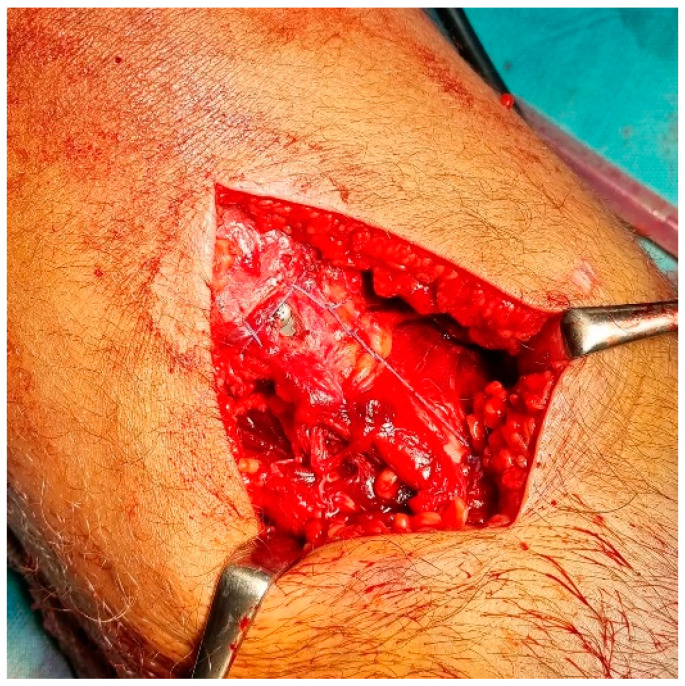
Intra-operative clinical images of the surgical technique.

**Figure 4:**
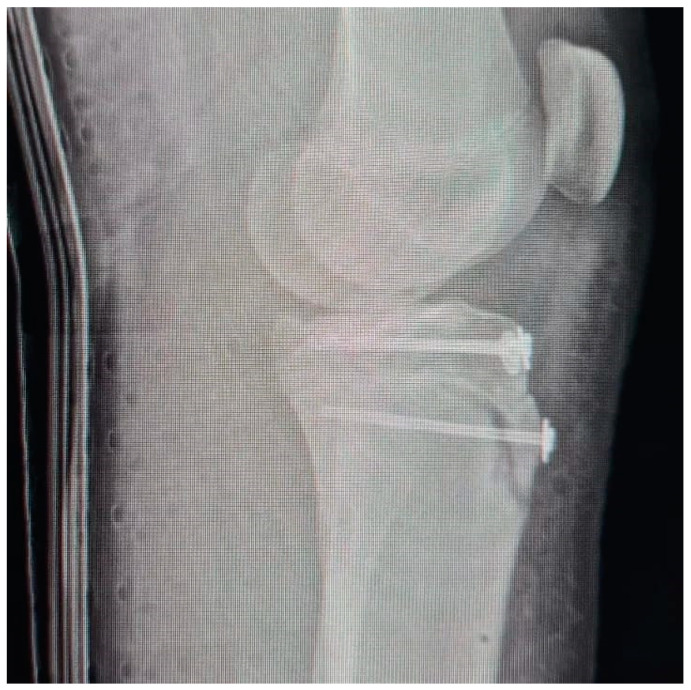
Post-operative X rays (Lateral view).

**Figure 5:**
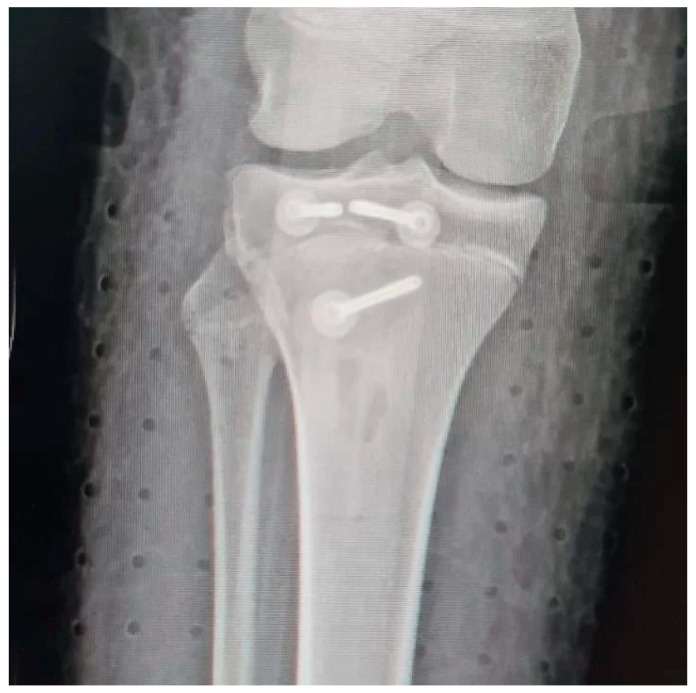
Post-operative X rays (antero-posterior view).

All patients had the same surgical treatment, an open reduction and internal fixation (ORIF) of the tibial apophysis with two or three cannulated screws (depending on the size and stability of the avulsed apophyseal fragment), followed by a primary tendon repair with a transosseous suture technique Krakow-like with Fiberwire (Figure 1, Figure 2, Figure 3, Figure 4 and Figure 5).

### 2.4. Rehabilitation Protocol

All patients followed this rehabilitation protocol:

- 0–3 weeks: Flexion allowed 0–45°, full weight bearing using a locking knee brace at 0° and isometric reinforcement.
- 4–6 weeks: flexion allowed 0–90°, full weight bearing using a locking knee brace at 0–60° and isometric reinforcement
- 7–12 weeks: Full flexion, full weight bearing, intensive muscle exercise
- From 12th week: Return to playing sport

### 2.5. Instrumentation

In this study, patients were asked to perform biomechanical physical tasks for the assessment of the outcomes of the surgical treatment in terms of muscle symmetry and strength. For this purpose, specific instrumentation was used: two force plates for the dynamic analysis, four sEMG probes for the muscular activity assessment, and a camera for the kinematic analysis.

The two force plates (60 cm × 80 cm, BTS PODIUM, BTS S.p.A., Milan, Italy) have a sampling rate of 1000 Hz and communicate with the computer using an Ethernet cable and the computer switch.

A camera (BTS VIXTA, BTS S.p.A., Milano, Italy) was used to conduct the video analysis. The camera was positioned in front of the force plates to acquire movement along both the frontal and sagittal planes with a sampling rate of 25 Hz and was connected to the computer using an Ethernet cable and a computer switch.

To collect the surface electromyographic signal (sEMG), the patients were equipped with four wearable probes for the (BTS FREEEMG 1000, BTS S.p.A., Milan, Italy), with a sampling rate of 1000 Hz. The probes were positioned on the target muscle (rectus femoris, vastus lateralis, vastus medialis, semitendinosus), chosen from the literature, according to the SENIAM recommendations. Adhesive Ag/AgCl electrodes (Kendall ECG Electrodes H124SG, Cardinal Health, Dublin, OH, USA) with an effective diameter of 10 mm and an inter-electrode distance of 20 mm (center to center) were employed to detect signals.

All instrumentation used was synchronized using the proprietary BTS SMART software (1.1).

### 2.6. Acquisition Protocol

The data were collected in the Laboratory of Functional Movement in the orthopedic department of the Bari Polyclinic, using a custom-made acquisition protocol. Following a state-of-the-art analysis, the target muscles and the motor tasks were selected. These tests were performed at 12-month follow-up after surgery for each patient.

The selected tasks were:

- The extension of the leg held for one minute to evaluate the maximum voluntary contraction: the patient was placed on a raised couch so that his or her feet did not touch the floor. The patient had to perform the extension of both legs (first the operated leg and then the healthy leg). The chosen leg was blocked using an obstacle that prevented full extension of the leg but allowed for maximum voluntary contraction; the operator was careful to ensure that the patient exerted maximum force equally throughout the entire minute.
- Single-leg squat (also known as “pistol squat”) repeated three times with a pause between each squat: the patient was positioned lateral to the camera. During the execution of the task, the subject must stand upright with feet shoulder-width apart, shift the weight onto one leg while lifting the other leg slightly off the ground, and extend the arms forward for balance. After setting the initial position, the subject is allowed to slowly lower their body by bending the knee of the supporting leg and extending the non-supporting leg straight out in front of them as they descend. The task requires continuing lowering until the supporting thigh is parallel to the ground, or as low as the subject is capable without compromising form, pause briefly at the bottom of the squat, and push through the heel of the supporting leg to return to the starting position.

The target muscles were the Rectus Femoris (RF), Semitendinosus (ST), Vastus Medialis (VM), and Vastus Lateralis (VL) muscles on both sides. The tasks were repeated twice: the first time to evaluate the performance using the operated leg and the second time using the healthy leg.

Each task was clearly outlined, and the researcher communicated the start and end times to all participants orally.

### 2.7. Biomechanical Data Processing

For the kinetic analysis, the force exchanged vertically between the foot and the ground was analyzed, i.e., the y-component of the force signal. The latter was filtered with a 4th-order low-pass Butterworth filter with a cut-off frequency equal to 15 Hz. In particular, the force was evaluated for one task, the single-leg squat, and the maximum force during the entire exercise was calculated, for both sides.

Both tasks were assessed using the sEMG signals. The sEMG signals were filtered using a 4th-order band-pass Butterworth filter with 10 Hz and 450 Hz cut-off frequencies. The sEMG signals were further processed, using a 2nd-order low-pass Butterworth filter with a cut-off frequency of 6 Hz, to calculate the signal envelope. Muscle activation was calculated using the Hodges and Bui method. Muscle activity was measured as the maximum value of the envelope (V), the percentage of activation relative to the entire cycle (%), and the presence/absence of pre-activation (yes/no).

For the analysis of the single squat, the three components of the force that the subject exchanged with the ground were filtered with a 4th-order low-pass Butterworth filter with a cut-off frequency of 15 Hz. The signal was segmented into the three phases that composed the task, so each phase comprised a single squat. For each squat, the maximum force exchanged with the ground was calculated and the average force between the three squats was calculated.

The biomechanical analysis of the force platform signal for the leg extension test in MVC was not possible given the setup for the acquisitions, which involved the use of a couch that impeded the evaluation of forces exchanged with the ground.

### 2.8. Statistical Analysis

Statistical analyses were conducted using SPSS for Windows software (v. 17.0; SPSS, USA). For the comparison of the percentage values (%) of activation of the muscle fibers of the muscles examined (VM, VL, ST, RF), a statistical analysis was performed using the "chi-square" evaluation. We assessed whether there was a statistically significant difference between the muscle groups of the operated limb and the groups of the healthy contralateral limb. *p* values < 0.05 are indicative of a statistically significant difference.

## 3. Results

The study consisted of 15 young patients undergoing surgical treatment for the fracture of the tibial apophysis with patellar tendon rupture with an average age 15.2 ± 1.1 years old. They were all male patients.

Table 1 shows the results of the questionnaire submitted to patients.

Table 2 and Table 3 show the results from the analysis of the muscular activity during the leg extension in MVC and the single squat task.

**Figure 6:**
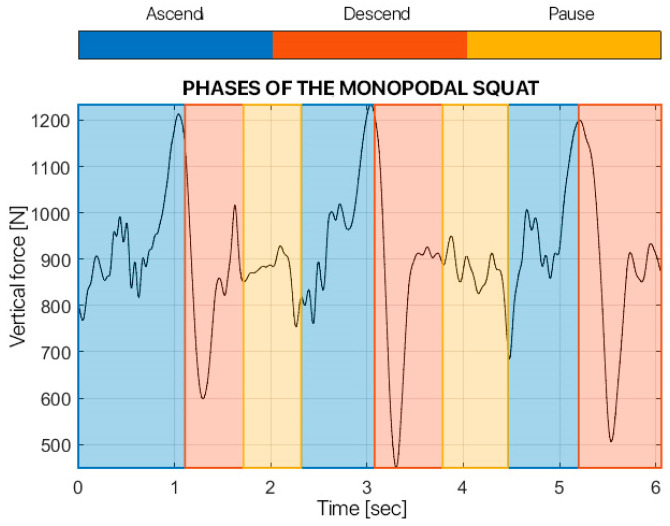
Phases of the monopodalic squat of surgically treated leg.

**Figure 7:**
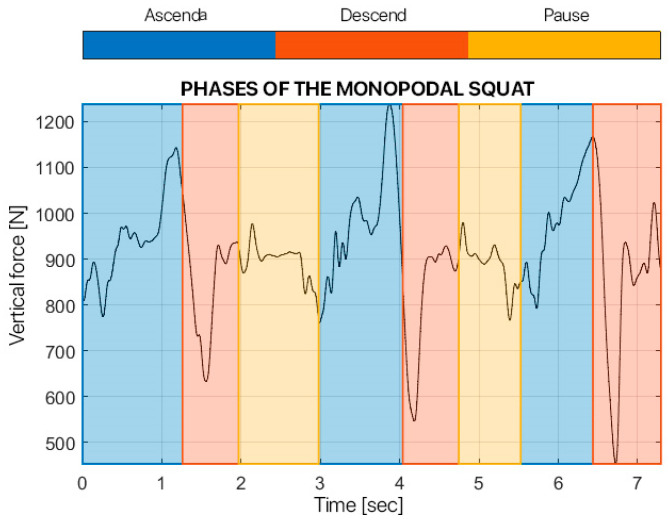
Phases of the monopodalic squat of healthy leg.

**Figure 8:**
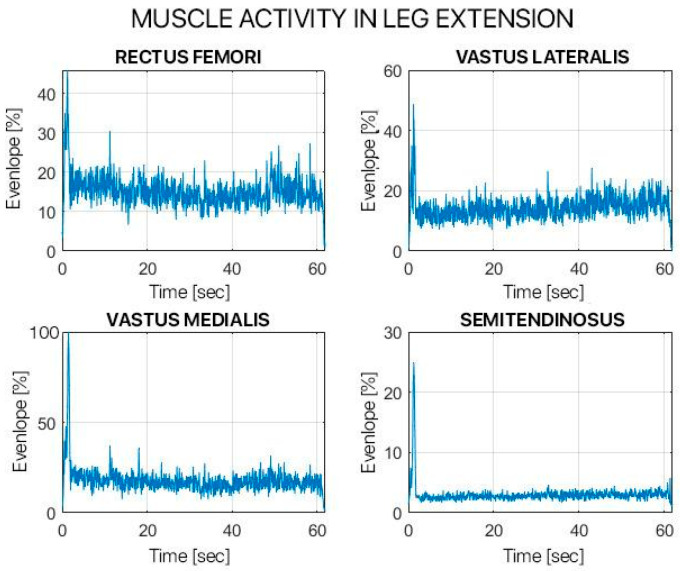
Muscle activity in leg extension of surgically treated leg.

**Figure 9:**
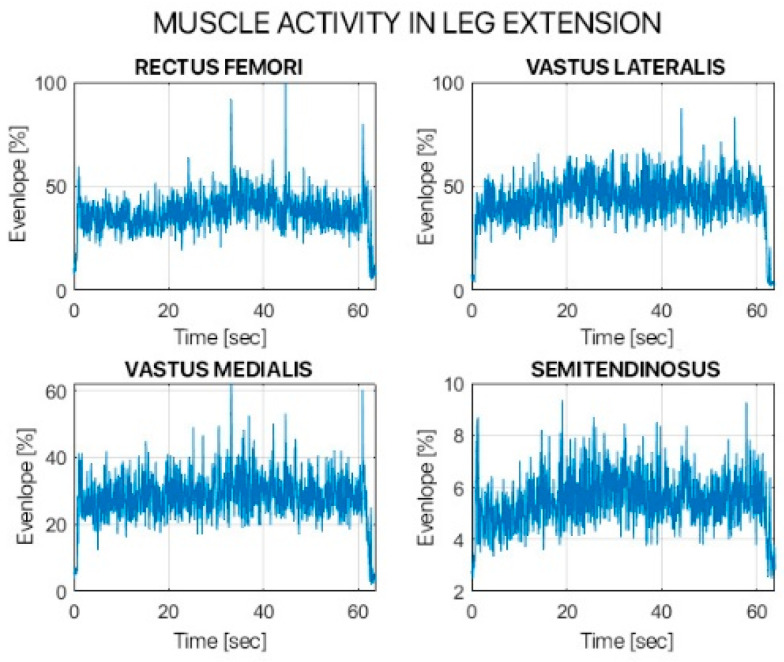
Muscle activity in leg extension in healthy leg.

Figure 6, Figure 7, Figure 8 and Figure 9 show the results from the dynamic evaluation of the single squat task. In particular, they show the maximum force the patient exchanged with the ground during the task.

Ten patients with a Level 9 TEGNER Scale returned to sport at the same level in 3.4 ± 0.6 months.

Among three patients with a Level 7 TEGNER Scale, two of these returned to sport at Level 9 and one at Level 7. Only one patient, with a Level 6 TEGNER Scale, returned to sport at a lower level (Level 4).

The statistical evaluation study comparing the activation of the muscle fascicles of the muscles examined highlighted that in our follow-up there was no statistically significant difference between the operated limb and the healthy one. This is true with the exception of two situations: VL during the leg extension exercise and RF during the single squat exercise (where the muscle activation of the healthy limb was statistically significantly higher than the operated one); and of the VM and ST during the single squat exercise (whereas the operated limb had statistically significantly higher values).

We reported two complications: one patient referred to knee stiffness and pain after surgical treatment, which has been solved when screws were removed.

In the other patient, we reported an aseptic loosening of implanted screws of the cannulated screws, which was treated with the removal of the screws and surgical curettage.

## 4. Discussion

Our study examined 15 young athlete patients who suffered an avulsion fracture of the tibial tubercle and who were treated surgically through ORIF of the avulsion fracture with a cannulated screws and washer, and a Krakow transosseous suture of the patellar tendon with Fiberwire. We subsequently followed the outcome of our patients with a 12-month follow-up, during which we were able to appreciate the recovery of activation and recruitment of the muscle fibers through bioengineering tests, comparing the restoration of strength of the operated limb with the healthy contralateral one, and their return to sport (RTS).

The kinetic analysis obtained by electromyographic signal of the examined muscles highlighted how 12 months after surgery there was a significant recovery in the percentage of muscle fiber activation during all the muscle tests performed. In some cases, especially for the vastus lateralis (VL), we even witnessed higher muscle activation than the healthy contralateral limb.

These data are in agreement with what has already been reported by Riccio et al., where no statistically significant differences were found when comparing terminal extension, terminal flexion, and total range of motion (ROM) between injured and healthy contralateral extremities at follow-up [10].

Another important element to analyze is that all the operated patients in our study have returned to practicing their sport. In the absence of further biomechanical investigations and in consideration of the fact that the patients were in a phase of both physical and sporting growth, our study does not allow us to demonstrate that they have returned to their pre-injury level. However, even with the results of the subjective questionnaires submitted, we can state that no patient reported complications or difficulties in returning to sporting activity compared to their pre-injury condition. Pretell-Mazzini et al. reported an RTS rate of 94% on a sample of 264 patients [11]. The complication rates reported in the literature for this type of surgical procedure are very low, as is the residual pain at the site of the operation. In fact, as reported by Haber et al., only 18% of patients reported residual pain on palpation, while only 10% reported pain during squats; only 1% of patients reported a reduction in ROM at maximum flexion and extension [12].

The data we collected and reported find confirmation and agreement with various previous scientific evidences, with functional outcomes following treatment of tibial tubercle avulsion fractures having been reported to be excellent in almost all cases treated surgically [1,13,14,15].

In a review on outcomes and complications published by Reyes et al., over a thousand patients undergoing open surgical treatment for avulsion fractures of the tibial tubercle were examined, reporting an excellent outcome rate in 95% of cases and with extremely low complication rates [16]. This underlines the safety and effectiveness of the surgical treatment for these avulsion fractures.

Based on current literature, devastating complications are rare, and even if they have been described, these are not typical complications for this operation, and so we cannot directly recommend paying attention to these particular complications. However, clinicians should stay vigilant to rule out acute vascular injuries or compartment syndrome [1,7,11,17].

## 5. Conclusions

Avulsion fracture of the tibial apophisis is an unusual injury in young athlete patients. Their surgical treatment, through open reduction and synthesis with a screw and a transosseous suture of the patellar tendon, has proven to be safe and effective. In our study we appreciated a total restoration of the strength and recruitment of the muscle fibers of the operated limb compared to the contralateral one, with an almost complete recovery of the muscle activation of the quadriceps and semitendinosus. Furthermore, all patients returned to their regular sporting activity at the preinjury level, without reported complications and with an excellent subjective satisfaction rate.

## Figures and Tables

**Table 1:** Study population and scores.

Variable	Mean ± St. Dev.
**Age (years)**	15.2 ± 1.1
**Time of the Injury (months)**	24.5 ± 7.5
**LYSHOLM**	95.5 ± 5.4
**TAS**	8.2 ± 2.7
**SF-12**	
PCS-12	54.6 ± 4.0
MCS-12	60.7 ± 2.0
**EQ 5D Y**	91.7 ± 13.3

**Table 2:** Percentage of muscular activation during leg extension.

Muscle	Operated Leg	Healthy Leg
**RF [%]**	**32.68**	32.92
**VL [%]**	26.82	**33.09**
**VM [%]**	23.92	25.59
**ST [%]**	6.17	5.97

**Table 3:** Percentage of muscular activation during single squat task.

Muscle	Operated Leg	HEALTHY LEG
**RF [%]**	20.89	23.91
**VL [%]**	**33.55**	**32.39**
**VM [%]**	29.70	25.54
**ST [%]**	13.89	9.81

## Data Availability

The original contributions presented in this study are included in the article. Further inquiries can be directed to the corresponding author.
